# Evaluation of Cross-Protection between G1a- and G2a-Genotype Porcine Epidemic Diarrhea Viruses in Suckling Piglets

**DOI:** 10.3390/ani10091674

**Published:** 2020-09-17

**Authors:** Yuhan Zhang, Yanjun Chen, Weifeng Yuan, Qi Peng, Fanfan Zhang, Yu Ye, Dongyan Huang, Zhen Ding, Longhua Lin, Houjun He, Qiong Wu, Deping Song, Yuxin Tang

**Affiliations:** 1 Department of Preventive Veterinary Medicine, College of Animal Science and Technology, Jiangxi Agricultural University, Nanchang 330045, China; zyhsh47@163.com (Y.Z.); cyj185704818@163.com (Y.C.); ywf519@outlook.com (W.Y.); jxaudkypq@hotmail.com (Q.P.); zfanfan0816@163.com (F.Z.); yy6157832@163.com (Y.Y.) ; huangdongyan@jxau.edu.cn (D.H.); dingzhenhuz@163.com (Z.D.); hehoujun@163.com (H.H.); lbls2005@sina.com (Q.W.); tang53ster@gmail.com (Y.T.); 2 Key Laboratory for Animal Health of Jiangxi Province, Jiangxi Agricultural University, Nanchang 330045, China; 3 National and Regional Joint Engineering Laboratory for Medicament of Zoonosis Prevention and Control, College of Veterinary Medicine, South China Agricultural University, Guangzhou 510642, China; 4 Jiangxi Engineering Research Center for Animal Health Products, Jiangxi Agricultural University, Nanchang 330045, China; 5 College of Animal Science and Technology, Jiangxi Agricultural University, Nanchang 330045, China; llh2918076946@163.com

**Keywords:** Porcine epidemic diarrhea virus, cross-protection, piglet, diarrhea, immunogenicity

## Abstract

**Simple Summary:**

Porcine epidemic diarrhea (PED), caused by PED virus (PEDV), is a devastating enteric disease in pigs worldwide. At least two genotypes (G1 and G2) and five subgenotypes (G1a, G1b, G2a, G2b, andG2c) of PEDV strains have been identified. To date, the reports on the antigenicity and immunogenicity of those viruses are limited and the results documented on cross-neutralization among different genotypes and/or subgenotypes of PEDV were inconsistent. This study aimed to observe the comparative pathogenicity and cross-protection between G1a and G2a PEDVs, and thus find a new insight into the antigenicity and immunogenicity of PEDVs. The results of the present study demonstrated that the G2a-based inactivated vaccine could provide sterilizing immunity against both highly virulent homologous and heterologous PEDV challenges. In contrast, the G1a-based inactivated vaccine could induce a sterilizing immune response against challenge of homologous strain CV777 and only provide partial protection for the challenge of a heterologous G2a PEDV CH/JX/01. The findings of this study might explain the underlying mechanism that severe PED and deaths still occurred among the neonatal piglets of which CV777-based PEDV vaccine were administered in China, and imply G2a-based PEDV vaccine used in this study might be a good vaccine candidate for PEDV which may provide solid protection against circulating highly virulent PEDVs.

**Abstract:**

To date, two genotypes, i.e., genotype 1 (G1) and genotype 2 (G2), of porcine epidemic diarrhea virus (PEDV) have been identified in swine, while the cross protection between the G2a and G1a subgenotypes is undetermined. Hence, in the present study, we attempted to observe a comparative pathogenicity and cross protection of G1a (CV777) and G2a (CH/JX/01) PEDVs. Initially pregnant sows were vaccinated twice with the two kinds of inactivated G1a- and G2a-based PEDV vaccines, respectively and the delivered neonatal piglets were challenged with prototype isolates of G1a and G2a PEDVs, and then the pathogenicity and cross-protection in neonatal piglets were observed. The results showed that CH/JX/01, a highly virulent and dominant G2a PEDV strain currently circulating in China had more severe pathogenicity in vitro and in vivo, and induced more strong immune responses, including higher titers of sIgA in maternal milk than that induced by CV777 PEDV, a prototype of G1a PEDV strain. All piglets from the sows immunized with CH/JX/01 could not only survive when challenged with the homologous PEDV, but also be fully protected when challenged with heterogenous G1a PEDV. In contrast, the piglets from the sows immunized with CV777 could be protected when challenged with homologous PEDV and only partially protected when challenged with heterologous G2a strain of PEDV (CH/JX/01). The findings of this study provide new insights into the pathogenicity, antigenicity, and immunogenicity of currently circulating wild type G2a PEDV, which might be valuable for the development of novel PEDV vaccine candidates with improved efficacy.

## 1. Introduction

Porcine epidemic diarrhea (PED), caused by porcine epidemic diarrhea virus (PEDV), is a devastating enteric disease in pigs, characterized with severe diarrhea, vomiting, and dehydration, especially with high morbidity and mortality (up to 100%) in suckling piglets [1]. PEDV is an enveloped, single-stranded, positive-sense RNA virus that belongs to the order *Nidovirales*, family *Coronaviridae* and genus *Alphacoronavirus* [2,3]. PED was first identified in the UK in 1971, and then spread to other European and Asian countries. In late 2010, severe outbreaks of diarrhea occurred in both non-vaccinated and CV777 vaccinated swine herds in China, attributing to highly virulent emerging PEDVs. Since then, the emerging variant PEDVs have been predominated in other Asian, North American, and European countries [4,5,6,7,8]. Subsequently, prototype PEDV variant strains with deletion(s) and/or insertion(s) in the spike (S) gene (so called INDEL in G1b subgenotype), with less severe clinical cases and lower case-fatality rate, was identified in the United States in 2013, and later reported in Asian and European countries [9,10,11,12,13,14].

Phylogenetic analyses based on the whole genome showed that all PEDV strains can be classified into two genotypes: Genotype I (G I or G1) and genotype II (G II or G2). G1 is represented by classical PEDV strains of CV777 and DR13, and the G2 compasses the highly virulent strains of variant PEDV [15,16]. The G1 PEDVs could be further divided into two sub-genotypes, i.e., G1a and G1b, respectively. The prototype PEDV of CV777 is a representative of G1a. And the G2 PEDVs are divided into three sub-genotypes, G2a, G2b, and G2c [17]. Genetic analyses revealed that the emerging highly virulent PEDVs being currently circulating in the field were different in various degrees from the classical PEDVs, including the vaccine strain of CV777 being used in China [4,18,19].

Until now, there have been considerable reports on the comparative pathogenicity and cross reactivity/protection among/between different sub-genotypes PEDV strains, including the observation in pathogenicity and cross-protection between G2a and G2b [20], G2b and G1b [21], G2b and G2c [22], G2b and G1 [23], and G1a and G1b [24]. Liu and colleagues reported a cross-protection between G2a and G2b strains, and found that G2a strain-based inactivated vaccine candidates had better efficacy to against both G2a and G2b strains than that of G2b-based candidates, shedding the lights on the development of an effective vaccine against the highly virulent PEDV strains [20]. However, the cross-protection between G2a and G1 in vivo, especially the protype G1a PEDV is still not documented. In this study, we isolated a highly virulent G2a PEDV strain from a diarrheal piglet, designated CH/JX/01, by which an inactivated cell culture-derived vaccine candidate was generated. Afterwards, the pathogenicity and cross-protection experiments of G2a strain CH/JX/01 towards G1a strain CV777 were performed by a passive immune protection approach, i.e., immunizing sows and then challenging suckling piglets fed with their mother milk.

## 2. Materials and Methods

### 2.1. Ethics Statement

The Jiangxi Agricultural University Institutional Animal Care and Use Committee approved the animal use protocol for this study (protocol number JXAU-AE-2017-11). All the procedures were carried out in accordance with The Care and Use Guidelines of Experimental Animals established by the Ministry of Agriculture of China.

### 2.2. Cells and Viruses

Vero-81 cells (ATCC^®^ CCL-81) were grown and maintained in Dulbecco’s modified Eagle’s medium (DMEM, Gibco, USA) supplemented with 10% fetal bovine serum (FBS, BI, USA), 100 U/mL penicillin, and 10 µg/mL streptomycin at 37 °C in a humidified atmosphere with the addition of 5% CO_2_. The highly virulent Chinese G2a PEDV strain, CH/JX/01 (GenBank accession number: KX058031) was isolated in our laboratory, and the G1a PEDV strain CV777 was generously provided by Dr. Zhen Li from Shanghai Academy of Agricultural Sciences. Both CH/JX/01 and CV777 were propagated in Vero-81 cells according to the previously described method [25,26]. Briefly, culture medium was removed from 90% confluent Vero-81 cell monolayers in T-25 culture flasks (Biofil, Guangzhou, China), and the cells were washed twice with sterile phosphate-buffered saline (PBS, 0.01M, pH 7.2, Solarbio, Beijing, China). Then, PEDV CH/JX/01 P10 stock was inoculated into the flask with a multiplicity of infection (MOI) of 0.1, supplied with 10 µg/mL final concentration of trypsin (Sigma, USA). After incubation for 1 h at 37 °C with 5% CO_2_, the supernatants were removed and 4 mL of virus growth medium containing 10 µg/mL trypsin was added, and was then cultured at 37 °C with 5% CO_2_. When more than 80% of the cells showed cytopathic effects (CPEs), the supernatants and cells were harvested, and stored at −80 °C until use.

### 2.3. Growth Kinetics of G2a and G1a Strains of PEDV and Indirect Immunofluorescence Assay (IFA)

Confluent Vero-81 cells in six-well plates were washed twice and then inoculated with 400 µL of viral supernatants containing CH/JX/01 or CV777 at a MOI of 0.1. After 1 h incubation at 37 °C with 5% CO_2_, the inoculum with unabsorbed viruses was removed and 2 mL of DMEM containing 10 µg/mL trypsin were added in each well, and then incubated at 37 °C with 5% CO_2_. The cell culture supernatants were collected at the time intervals of 4, 8, 12, 16, 20, 24, 28, 32, 36, 40, 44, and 48 h(s), respectively, and titrated by the median tissue culture infectious dose (TCID_50_) according to the protocol described previously [27].

Vero-81 cells were cultured in 12-well plates containing sterile covering slides and incubated with CV777 and/or CH/JX/01 at a MOI of 0.1, respectively. After the virus was inoculated, the slides were taken out at 3 h, 6 h, 12 h, 24 h, 36 h, and 48 h, and then fixed with pre-cooled glutaraldehyde for 10 min, followed by rinsing with 0.01 M pH 7.4 PBS. The fixed cells were allowed to dry on air for at least 10 min. Then, 0.5% *v*/*v* triton X-100 (Sigma-Aldrich, Shanghai, China) was applied to each well for 10 min. After washing three times with PBS, the cells were overlaid with 5% w/v bovine serum albumin (BSA) in PBS and incubated at 37 °C for 30 min. The rabbit anti-PEDV antibody made by our lab was used as the first antibody with a 1:100 dilution in PBS. After 1 h incubation at 37 °C, the cell monolayers were rehydrated by rinsing three times with PBS. Coverslips from each well were stained using 100 μL 1:2000 diluted goat anti-rabbit immunoglobulin G (IgG) conjugated with fluorescein isothiocyanate (FITC; Sigma-Aldrich, St. Louis, MO, USA) and incubated for a 1 h at 37 °C. FITC-stained cells were then rinsed with PBS, and immediately examined under a fluorescence microscope (Axio Observer A1, CARL ZEISS, Oberkochen, Germany) [28].

### 2.4. Animals

Six Large White x Duroc crossbred pregnant sows at 70 to 74-day of gestation were purchased from a commercial pig farm, which had neither previous outbreak of PED nor vaccination of PEDV vaccines during the recent two years. Initially, all sows had been tested for antigen negative of PEDV by an established real-time PCR assay [26], and sero-negative for PEDV antibody by a commercial ELISA kit (Biovet, Calgary, AB, Canada). Each sow was housed in a separate room, farrowed routinely in our animal facility in biosafety level-2 laboratory.

### 2.5. Vaccination and Challenge

**Figure 1:**
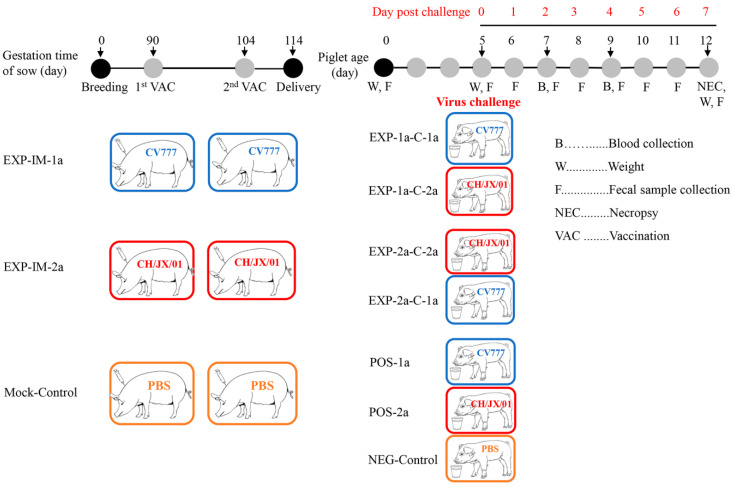
Experimental design and sample collection. The top line indicates the day post porcine epidemic diarrhea virus (PEDV) challenge, the second line on the left indicates the gestation stage of sows, and the right line indicates the age of neonatal piglets.

The eighteenth passage (P18) of cell culture-adapted PEDV strain CH/JX/01 with a titer of 10^7.0^ TCID_50_/_mL_ were inactivated using binary ethyleneimine (BEI) at a final concentration of 0.2% incubating at 30 °C for 72 h. After inactivation, the remaining BEI was neutralized by the addition of 20% sodium thiosulfate. To assure a complete inactivation of the virus, a standard procedure was employed by growing the preparation in Vero-81 cell cultures. Subsequently, the inactivated virus suspension was 1:1 *v*/*v* mixed with a water-in-oil-water adjuvant, MONTANIDE ISA 201 VG (SEPPIC, Shanghai, China) according to manufacturer’s instructions, and the prepared vaccines were stored at 4 °C until use. The detailed experiment design is shown in Figure 1. Six PEDV antigen- and sero-naïve sows were assigned randomly to three groups: (1) Group 1, designated as EXP-IM-2a, sows in this group were intramuscularly vaccinated with a dose of 2 × 10^7.0^ TCID_50_ CH/JX/01 (in 4 mL volume) inactivated vaccine in the neck; (2) group 2, designated as EXP-IM-1a, sows in this group received 4 mL (2 × 10^7.0^ TCID_50_) of an inactivated CV777-based vaccine intramuscularly into the neck; and (3) group 3, named as Mock-Control, sows in this group were sham-vaccinated intramuscularly in the neck with 4 mL of PBS. All sows were firstly immunized at 90th pregnant day and re-immunized at 104th pregnant day.

Healthy neonatal suckling piglets born with the body weight of >1.2 kg were kept for this experiment, and used for virus challenge at 5 d of age. The piglets from group EXP-IM-2a were randomly divided into two groups (five piglets/group), and orally challenged with prototype CV777 (1 × 10^5.0^ TCID_50_ per piglet) and CH/JX/01 (1 × 10^4.2^ TCID_50_ per piglet), respectively. Similar operations were carried out on piglets from EXP-IM-1a as indicated in Figure 1. Piglets from Mock-Control group were randomly divided into three groups (five piglets each group), and orally challenged with prototype CV777, CH/JX/01, and PBS, respectively (Figure 1). The groups were housed in separate rooms after challenge and were observed three times daily for the first 7 days post challenge (dpc). All piglets were weighted at dpc 0 and dpc 7. Fecal score (FS) was recorded twice daily after challenge, and was scored under the criteria of 0 = solid, 1 = pasty, 2 = semi-liquid, 3 = liquid, respectively. Rectal swabs were collected twice a day from 0 dpc to 7 dpc for PEDV fecal shedding. The RNA was extracted of rectal swabs using TRIzol plus (TaKaRa, Dalian, China), and the titers/copies of PEDV RNA were quantified by an established TaqMan real-time RT-PCR in our lab [29]. Samples were considered negative when no signal was observed within 40 amplification cycles. All of the lived piglets at 7 dpc were euthanized for gross and histopathology examination. Milk samples were also collected from sows at the 7th day post parturition, and whey was prepared from milk samples following previously described protocols [30]. Briefly, components of fat globules, casein micelles, and cells, which are known to interfere with immunological assays, were removed, then whey was used to determine sIgA responses by a commercial indirect ELISA kits (Yoyoung, Guangzhou, China). The levels of Tumor necrosis factor-α (TNF-α) in piglet serum samples in all groups were tested with commercial ELISA kit following the manufacturer’s instructions (Invitrogen, Frederick, MD, USA).

### 2.6. Gross and Histopathological Examinations

At necropsy, both intestine and other major organs were examined. Duodenum (5 cm distal to the pylorus), jejunum (three samples taken at 40–60 cm intervals), and ileum (5 cm anterior to the ileocaecal valve) were collected. After 48 h fixation in 4% paraformaldehyde, tissues were serially dehydrated with 30%, 50%, 70%, 95%, and 100% ethanol, cleared in xylene, embedded in paraffin wax, and sectioned at 4–6 μm thickness. After dewaxing in xylene and serially rehydrating with 100%, 95%, and 70% ethanol, tissue sections were stained with hematoxylin and eosin (HE, Sigma-Aldrich, Shanghai, China) and then examined by conventional light microscopy. For each tissue section, at least ten villi and crypts were measured using a computerized image system with villous height and crypt depth (VH:CD) ratios calculated as previously described procedures [31].

### 2.7. Statistical Analysis

A randomized design was applied in the study, and the replicate was defined as the experimental unit. Statistical analysis was performed using SPSS software 25.0 (IBM Corporation, Armonk, NY, USA). The data of this study were assessed for the normal (Gaussian) distribution by using the Shapiro–Wilk Test in SPSS before performing the statistical analysis. Statistical analysis of the growth titers of PEDV strains belonging to different subgenotypes and body weight changes in piglets was performed using paired-samples Student’s *t* test. A Kaplan–Meier survival curve with log-rank test was used to compare piglet survival rate among the experimental groups. Statistical analysis of the indices of villus height, crypt depth, thickness and the titer of sIgA was carried out by one-way analysis of variance (ANOVA) and significant differences among group means were determined using the least significant difference (LSD) test. Statistical analysis of the relative express level of cytokines was performed by using the Tukey test. Data are presented as the mean ± standard error of the mean (SEM). A *p*-value of <0.05 was set as the statistically significant level.

## 3. Results

### 3.1. Comparative Growth Kinetics of G2a and G1a PEDV Strains In Vitro

**Figure 2:**
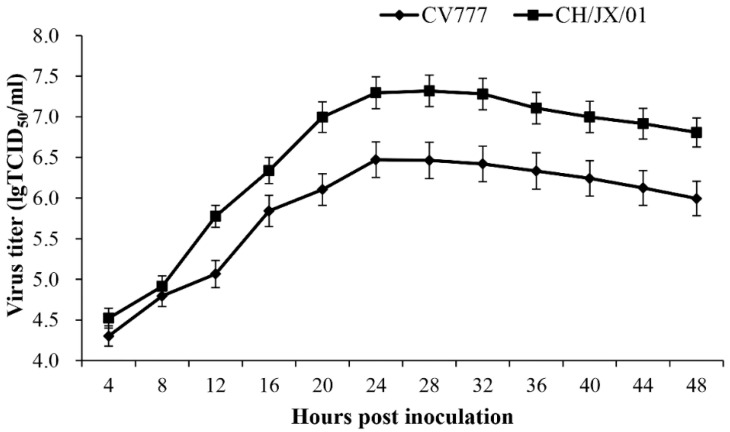
The one-step growth curve of CH/JX/01 and CV777 in Vero-81 cells.

**Figure 3:**
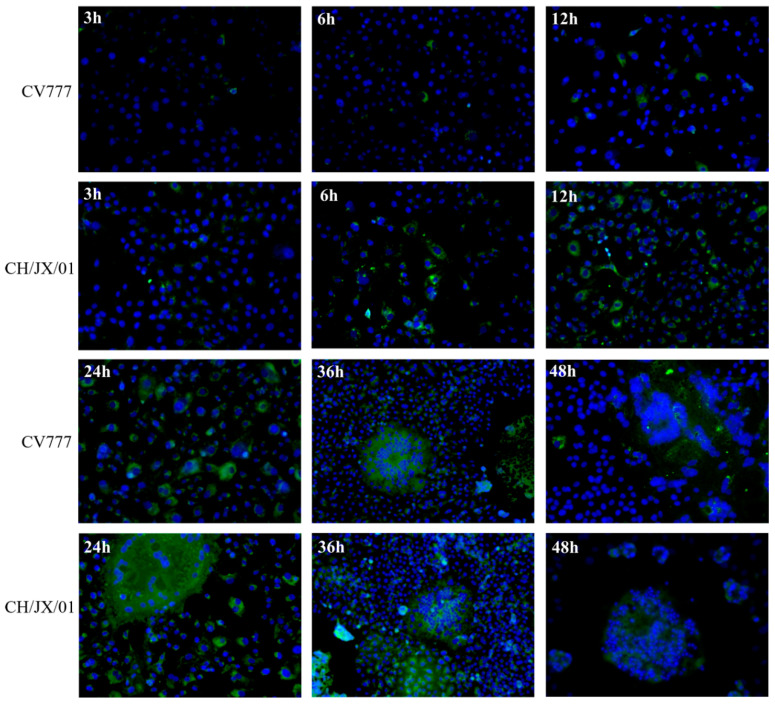
Indirect immunofluorescence assay to detect infection of PEDV strains CV777 and CH/JX/01 in Vero-81 cells.

To compare the infectivity of CH/JX/01 and CV777 in vitro, the growth performance was assessed by growth kinetics and IFA. The titers of both viruses reached the plateau at 24 h post infection (hpi) in cell culture supernatants, while the TCID_50_ of CH/JX/01 (10^7.32^ TCID_50_/_mL_) was over seven times as that of CV777 (10^6.47^ TCID_50_/_mL_) (Figure 2). The IFA results indicated that PEDV N protein was present in the cytoplasm of Vero-81 cells. Both CH/JX/01 and CV777 induced an apparent and typical CPE in Vero-81 cells, including cell fusion, cell detachment, and multinucleated giant cell formation. The fluorescence signals of CH/JX/01-infected cells appeared as earlier as 3 h post inoculation (hpi) and the signals were stronger than those of CV777-infected cells, which indicated CH/JX/01 was more aggressive and pathogenic than that of CV777 (Figure 3). At the same time point of post inoculation, the syncytia of CH/JX/01-infected cells appeared earlier and in greater numbers than those of CV777-infected cells, suggesting that CH/JX/01 proliferated faster and was supposed to be more pathogenic than CV777. The results of IFA were consistent with the results of growth kinetics.

### 3.2. Clinical Assessment of Piglets under Virus Challenge

Before challenge, all piglets were healthy and had no any clinical signs, and were antigen/antibody negative for PEDV. After challenge, clinical symptoms, fecal scores (FS), body weight, morbidity, and mortality were observed and recorded (Table 1). Piglets in POS-2a group were firstly observed watery diarrhea (FS = 3) in 12 h post challenge (hpc), diarrhea lasted from 12 to 96 hpc, and all piglets in this group were died; within the 96 hpc, acute vomiting was noted in 3 piglets, and 21 cases of watery diarrhea (21/31, 67.74%) were recorded, 5 cases of diarrhea with semi-liquid feces (FS = 2, 16.13%), 2 cases of mild diarrhea (FS = 1, 6.45%), and 3 cases of solid feces (FS = 0, 9.68%) were observed. The CV777-vaccinated (EXP-IM-1a) and CH/JX/01-vaccinated piglets (EXP-IM-2a) could resist the challenges of homologous viruses, while one piglet showed pasty feces from 60 to 84 hpc in group EXP-2a-C-2a, and one piglet had pasty feces from 72–96 hpc in group EXP-1a-C-1a. All piglets from EXP-IM-1a challenged with CH/JX/01 (EXP-1a-C-2a) developed watery diarrhea from 48–144 hpc, and 60% of the piglets died in the first week. No diarrhea was observed in piglets from NEG-Control and EXP-2a-C-1a in the challenge experiments. In addition, the duration of diarrhea negatively correlated to the body weight gain in the first week for all piglets. Compared to the weight gain in the first 7 days of NEG-Control piglets (1.41 ± 0.20 kg), no significant difference was observed in piglets in groups EXP-1a-C-1a (1.31 ± 0.38 kg), EXP-2a-C-2a (1.21 ± 0.11 kg), and EXP-2a-C-1a (1.25 ± 0.64 kg). In contrast, significant weight losses were observed in piglets from groups EXP-1a-C-2a (−0.70 ± 0.19 kg, ANOVA, *p <* 0.05), POS-1a (0.80 ± 0.20 kg, *p <* 0.05), and POS-2a (−0.35 ± 0.21 kg, ANOVA, *p <* 0.01) (Table 1 and Supplementary Table S1).

### 3.3. Gross Lesions, Histopathology, and Immunohistochemistry Staining

Clinically, the piglets in groups EXP-1a-C-2a, POS-1a, and POS-2a were all extremely emaciated and severely dehydrated as evidenced by sunken eyes, inelasticity of the skin, and tacky subcutaneous tissues. For piglets in POS-2a, watery diarrhea observed as early as 12 hpc, severe dehydration showed within 24 h after watery diarrhea, and all of the piglets died within 6 dpi. The surviving piglets were euthanized at 7 dpc for the observation of gross and histopathological lesions. Necropsy indicated that the small intestine walls were transparent and thin in all challenged groups, with POS-2a being the most severe. In some piglets, the intestinal lumens were filled with large amounts of liquid intestinal content. No gross and histopathological lesions were observed in the piglets of NEG-Control group.

**Figure 4:**
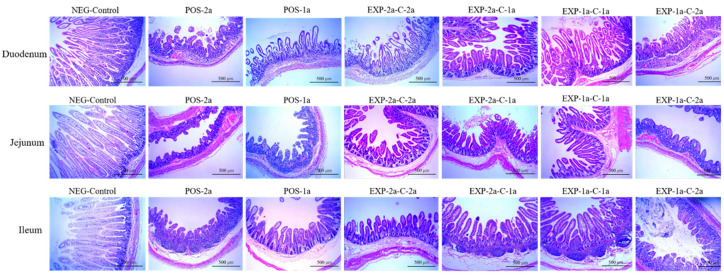
Hemotoxylin and eosin-stained tissue sections of small intestine in piglets challenged by PEDV strains CV777 and/or CH/JX/01 (×100).

Microscopic lesions were seen in the PEDV infected piglets, pigs which had mild to severe atrophic enteritis at 7 dpc. Shortening, blunting and fusion of the villi, and occasionally, vacuolization and exfoliation of enterocytes were noted. Microscopic lesions observed in the CH/JX/01-infected piglets (POS-2a) were more severe than those observed in CV777-inoculated piglets (POS-1a). The VH:CD ratio in duodenum were 0.99 in piglets in POS-2a while 1.81 in piglets in POS-1a (Figure 4 and Table 2). In the immunization-challenge groups (EXP-2a-C-2a, EXP-2a-C-1a, EXP-1a-C-2a, EXP-1a-C-1a), the microscopic lesions in EXP-1a-C-2a were more severe than those observed in EXP-2a-C-1a, with more shortened, blunted villi in duodenum and jejunum. The VH:CD ratios ranged between 1.55 and 1.97 in duodenum, jejunum, and ileum. No significant gross and microscopic lesions were noted in NEG-Control, and the VH:CD ratios were 10.62, 14.27, and 14.55 in duodenum, jejunum, and ileum, respectively (Figure 4 and Table 2).

**Figure 5:**
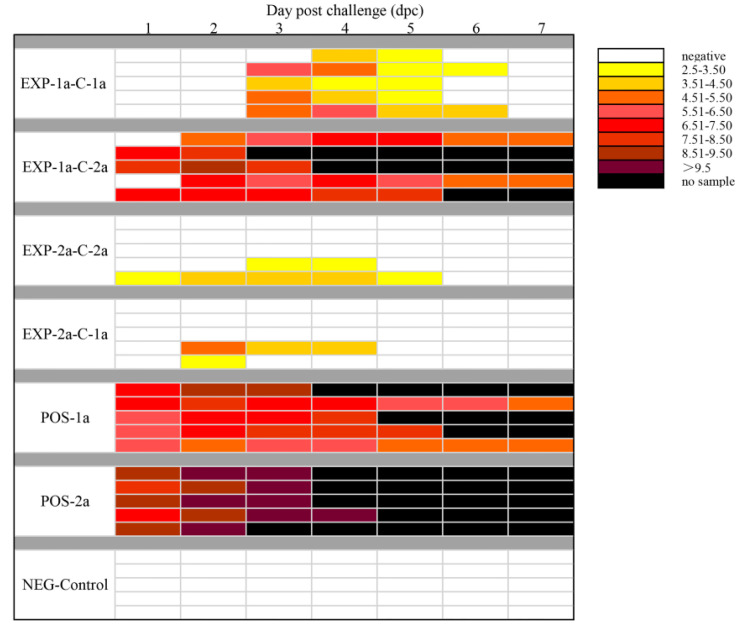
PEDV shedding patterns for all challenged piglets over time. For each group a line corresponds to the quantity of virus shed in rectal swabs for a single piglet from dpc 1 to dpc 7 is shown on the right side of the heatmap.

Virus shedding in feces in all experimental piglets was examined daily by PEDV-specific TaqMan real-time RT-PCR. All piglets were negative for PEDV RNA in fecal swabs on 0 dpc and NEG-Control piglets remained negative for the duration of the 7 days post challenge. Fecal shedding was detected in 5/5 POS-1a and 5/5 POS-2a piglets within 1 dpc, and the titers of PEDV RNA in piglets in POS-2a were magnitude higher than that in piglets within POS-1a. Compared to EXP-1a-C-1a, piglets in EXP-1a-C-2a had more frequent virus shedding and the titers of the virus were higher. Among piglets in EXP-2a-C-2a, a piglet showed pasty was found virus shedding from dpc 1 to dpc 5 (Figure 5 and Supplementary Table S2).

### 3.4. Protective Effects of Different Levels of Maternal Antibodies on Piglets

**Figure 6:**
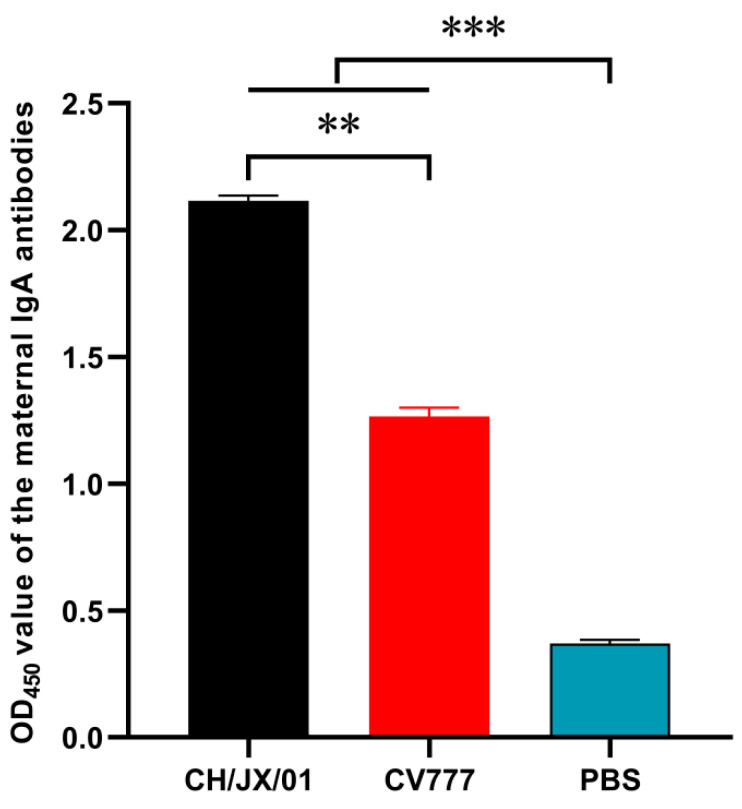
Group mean anti-PEDV sIgA response in maternal milk. Data presented as mean group ELISA sample-to-positive (S/P) ratios ± SEM. The significance level was set to ** indicates 0.001 < *p* ≤ 0.01, and *** indicates *p* ≤ 0.001.

**Figure 7:**
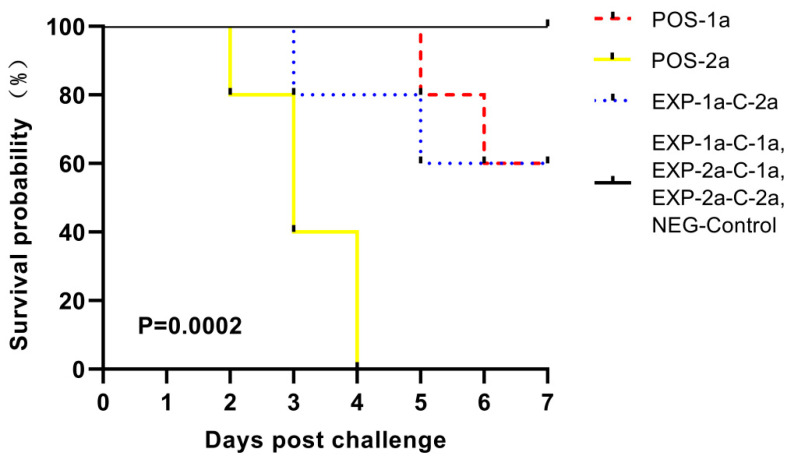
Kaplan–Meier curves for mortality of piglets orally challenged by CV777 or CH/JX/01.

The colostrum antibody responses induced by vaccinated CH/JX/01 and CV777 were assessed by a commercial indirect-ELISA kit. As shown in Figure 6, the CH/JX/01 and CV777 strains were all induced specific sIgA responses, while the titer of the sIgA stimulated by CH/JX/01 was significantly higher than that induced by CV777 at 7 days post parturition (*p* < 0.01). The protective effects of two maternal antibodies against challenge with virulent homologous and heterologous virus (CH/JX/01, CV777) were set by the observation of clinical signs and mortality of the piglets during the 7-day observation period. The results indicated that maternal antibodies induced by CH/JX/01 could protect 100% (5/5) piglets against both CH/JX/01 and CV777 challenge, while the maternal antibodies induced by CV777 protected only 40% (2/5) piglets against CH/JX/01. The survival curves were in accordance with the results of cross-protection and maternal sIgA antibodies (Figure 7). No piglets in group POS-2a survived under CH/JX/01 challenge.

### 3.5. Serum TNF-α Concentration in Pigs

**Figure 8:**
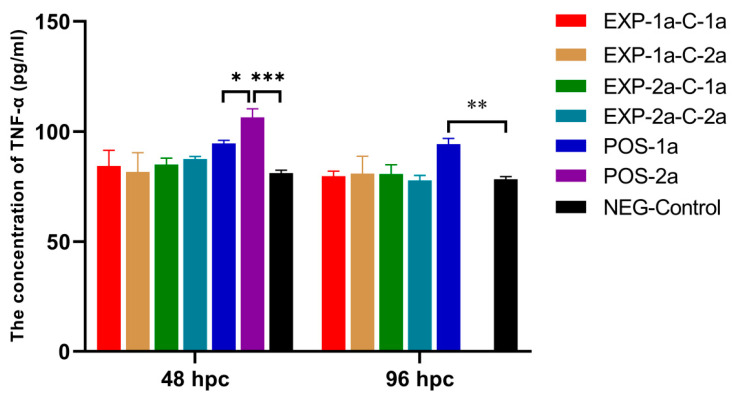
Levels of the cytokine Tumor necrosis factor-α (TNF-α) in all piglets at 48 and 96 hpc. The mortality of POS-2a was more than 40% in 96 hpc, the levels TNF-α were not detected; * indicates *p* < 0.05, ** indicates 0.001 < *p* ≤ 0.01, and *** indicates *p* ≤ 0.001.

The expression of TNF-αwas tested in the sera of all piglets. The concentrations of TNF-α in groups POS-2a and POS-1a were significantly higher than the NEG-Control group at 48 and 96 hpc (*p* < 0.05, Figure 8). No significant change of TNF-α was found in piglets within groups EXP-1a-C-1a, EXP-1a-C-2a, EXP-2a-C-2a, and EXP-2a-C-1a.

## 4. Discussion

The continued epidemic of PED has caused huge economic losses to the pig industry around the world. In China, the mortality of piglets within 7-day age associated with PEDV infection could reach 80% to 100% [18]. In USA, PEDV infection has reduced domestic pig population by nearly 10% during 2013 to 2014 [32]. Epidemiologic survey and phylogenetic analysis indicated that most of the PEDV strains currently circulating in the field were variants of PEDV, which are highly pathogenic and caused severe morbidity and mortality in piglets [31,33,34]. The highly virulent PEDV strains were genetically different from the classical PEDV strains [17]. As reported, there are many nucleotide variations between G1 and G2 PEDV strains, especially in spike gene, which may lead to the failure of traditional attenuated vaccines being used in Asia [15]. In our study, biological characteristics between CH/JX/01 and CV777 suggested that CH/JX/01 grew faster and induced more severe CPE in cells and caused more severe diarrhea and death in piglets, indicating that the CH/JX/01 were more virulent than that of classic PEDV CV777.

Several subgenotypes of field PEDV-based vaccines are being used in China, but the efficacies are tremendously variable. Many pig herds vaccinated with classical PEDV vaccines failed to provide solid protection against highly virulent PEDVs [19,32]. So, it is essential to investigate the cross-neutralization of different subgenotypes of PEDV circulating in the field and find novel vaccine candidates with improved efficacy. Since it’s costly to use pregnant sows as experimental models, many studies on the cross-protections evaluation use neonatal or weaning piglets. In practice, most infections were contracted to piglets within 7 days old due to the lack of adaptive immunity against PEDV. For suckling piglets, the maternal antibodies provide the primary protection against the PEDV infection. In this study, to evaluate the homologous and heterologous reactivities of G1a and G2a PEDVs, we initially immunized pregnant sows twice with inactivated G1a and G2a PEDV vaccines and then challenged piglets with the prototype strains of G1a PEDV strain CV777 and field very virulent G2a PEDV strain CH/JX/01, respectively. The sows vaccinated with the inactivated CH/JX/01 had a stronger sIgA responses in milk than that of CV777. It has been shown that sIgA levels in maternal milk correlated with sIgA measured in sera of suckling piglets [35]. Goede and colleagues found that previous infection of sows with a mild virulence PEDV strain could confer protection on piglets against high virulent PEDV strain [16]. Lin et al. reported that vaccinated inactivated PEDV could provide piglets against homologous challenge [36]. So, the piglets fed by sows immunized with CH/JX/01 could obtain more anti-PEDV sIgA in milk, and thus passively protected by those sIgA originated from their mothers. When challenged with very virulent strain of CH/JX/01, piglets in groups POS-2a had earlier disease onset, higher diarrhea index and earlier death time than those infected with CV777 strain, which indicated CH/JX/01 had higher pathogenicity than that of CV777.

Results from studies on cross-reactivity, cross-neutralization, or cross-protection between different subgenotypes of PEDV were various. Researchers evaluated cross-reactivity between G2b non S-INDEL and G1b S-INDEL PEDVs using convalescent sera from pigs, and confirmed the serological cross-reactivity between the two subgenotype strains in vitro [11]. A study presented by Lin et al. showed that previously infected with S-INDEL PEDV could just protect 81.25% (13/16) protection on challenge by virulent non S-INDEL PEDV [37]. In the present study, piglets from sows vaccinated with G2a and G1a PEDVs could sustain the challenges of homologous viruses, and provide cross-protection to G1a strain of PEDV. However, sows immunized with G1a PEDV could not provide cross-protection to the G2a strain virus challenge. This implicated that G2a PEDV might provide immunity to both G2a and G1a PEDVs. Liu and colleagues reported that the G2a strain-based inactivated vaccine candidates are more promising than G1b-based PEDV candidates for the development of an effective vaccine against the current highly virulent pandemic PEDV strains [20]. So, the G2a PEDV strain might be a better vaccine candidate strain for vaccine development.

Proinflammatory and anti-inflammatory cytokines (e.g., TNF-α and IL-6, IL-10, IL-12, and IL-22) are produced to mediate various inflammatory responses and exert antiviral effects [38,39,40]. Previous studies have shown that TNF-α can induce a striking increase in the number of intra-alveolar neutrophils and their phagocytic capacity against various viruses. In addition, TNF-α induces apoptosis in cells infected with a virus [41,42]. The levels of serum TNF-α were in accordance with the symptoms of piglets. Piglets in group POS-2a with most severe diarrhea and death had the highest TNF-α level, while the levels in piglets within groups EXP-1a-C-1a, EXP-1a-C-2a, EXP-2a-C-2a, and EXP-2a-C-1a were not significantly increased. The reason might due to the maternal antibody neutralized part of the viruses and suppressed the inflammatory responses. However, further study is needed to explore the interactions between PEDV and cellular cytokines.

## 5. Conclusions

The highly virulent G2a PEDV strain CH/JX/01 induced stronger CPE, grew faster in Vero-81 cells, and induced higher sIgA level in maternal milk in vivo than those in G1a PEDV strain CV777. Furthermore, vaccination with CH/JX/01 could not only protect the homologous challenge, but also showed a full cross-protection to heterogenous PEDV strain CV777 challenge. The findings of this study provide new insights into the pathogenicity, antigenicity, and immunogenicity of currently circulating wildtype G2a PEDV, which might be valuable for the development of novel PEDV vaccine candidates with improved efficacy.

## Figures and Tables

**Table 1:** Clinical observations of piglets in the first week post challenge.

Challenge Group	Body Weight Gain in 7 dpc (kg)	Onset of Diarrhea (hpc)	Fecal Scores Accumulation and Ratio				Mortality
			FS = 3	FS = 2	FS = 1	FS = 0	
EXP-1a-C-1a	1.31 ± 0.38	None ^a^	0 (0)	0 (0)	3 (4.29%)	67 (95.71%)	0
EXP-1a-C-2a	−0.07 ± 0.19 *	48–144	19 (38.78%)	11 (22.45%)	10 (20.41%)	9 (18.37%)	60%
EXP-2a-C-2a	1.21 ± 0.11	None ^b^	0 (0)	0 (0)	5 (7.14%)	65 (78.57%)	0
EXP-2a-C-1a	1.25 ± 0.64	None	0 (0)	0 (0)	4 (5.71%)	66 (94.29%)	0
POS-1a	0.80 ± 0.20 *	48–144	28 (50.00%)	12 (21.43%)	10 (17.86%)	6 (10.71%)	60%
POS-2a	−0.35 ± 0.21 **	12–96	21 (67.74%)	5 (16.13%)	2 (6.45%)	3 (9.68%)	100%
NEG-Control	1.41 ± 0.20	None	0 (0)	0 (0)	2 (2.86%)	68 (97.14%)	0

Note: * indicates 0.01 < *p* value < 0.05, ** indicates 0.001 < *p* value < 0.01. FS, fecal score; FS = 0, 1, 2, and 3 corresponded to solid, pasty feces, diarrhea with semi-liquid feces, and watery diarrhea with liquid feces, respectively. ^a^: A piglets showed pasty feces from 72 to 120 h post challenge (hpc); ^b^: A piglet showed pasty feces from 60 to 108 hpc. dpc: days post challenge.

**Table 2:** Effects of different treatment on villus height, crypt depth, and thickness of intestine wall of piglets.

	Group	Villus Height (VH, μm)	Crypt Depth (CD, μm)	VH:CD Value	Intestine Wall Thickness (μm)
Duodenum	EXP-1a-C-1a	754.35 ± 39.96 ^C^	156.73 ± 6.78 ^bcAB^	4.81	356.82 ± 22.25 ^B^
	EXP-1a-C-2a	214.33 ± 10.52 ^DE^	108.8 ± 6.76 ^dC^	1.97	317 ± 23.31 ^B^
	EXP-2a-C-2a	970.23 ± 61.35 ^B^	175.89 ± 11.41 ^abAB^	5.52	319 ± 12.17 ^B^
	EXP-2a-C-1a	958.64 ± 30.88 ^B^	169.26 ± 13.12 ^abAB^	5.66	310.15 ± 16.61 ^B^
	POS-1a	337.18 ± 54.45 ^D^	186.22 ± 6.42 ^aA^	1.81	307.04 ± 29.65 ^B^
	POS-2a	134.02 ± 9.93 ^E^	135 ± 4.24 ^cdBC^	0.99	134.72 ± 17.78 ^C^
	NEG-Control	1208.75 ± 56.74 ^A^	113.83 ± 8.71 ^dC^	10.62	751.5 ± 26.05 ^A^
Jejunum	EXP-1a-C-1a	785.17 ± 20.46 ^bcB^	146.62 ± 6.41 ^aA^	5.36	215.43 ± 5.46 ^cBC^
	EXP-1a-C-2a	180.33 ± 10.3 ^dC^	105.28 ± 6.37 ^bBC^	1.71	280.28 ± 19.16 ^bBC^
	EXP-2a-C-2a	965.17 ± 50.79 ^bB^	133 ± 6.99 ^aA^	7.26	215.15 ± 14.83 ^cBC^
	EXP-2a-C-1a	708.5 ± 50.17 ^cB^	146.78 ± 4.79 ^aA^	4.83	220.08 ± 9.78 ^cBC^
	POS-1a	140.3 ± 6.64 ^dC^	143.24 ± 10.4^a A^	0.98	174.03 ± 15.36 ^cC^
	POS-2a	162.9 ± 12.04 ^dC^	135.91 ± 9.66 ^aA^	1.20	157.66 ± 6.43 ^cC^
	NEG-Control	1333.92 ± 93.33 ^aA^	93.5 ± 3.33 ^bC^	14.27	562.83 ± 26.79 ^aA^
Ileum	EXP-1a-C-1a	1040.2 ± 20.01 ^B^	164.82 ± 8.97 ^aA^	6.31	299.48 ± 41.52 ^bcCD^
	EXP-1a-C-2a	175.56 ± 10.4 ^D^	113.56 ± 7.33 ^bC^	1.55	314 ± 12.07 ^bcCD^
	EXP-2a-C-2a	380.58 ± 15.48 ^C^	162 ± 9.08 ^aA^	2.35	293.98 ± 37.04 ^bcCD^
	EXP-2a-C-1a	382.61 ± 53.40 ^C^	127.89 ± 6.58 ^bC^	2.99	371.56 ± 23.87 ^aCD^
	POS-1a	140.26 ± 8.26 ^D^	165.41 ± 12.26 ^aA^	0.85	289.91 ± 20 ^bcCD^
	POS-2a	124.72 ± 10.95 ^D^	128.37 ± 6.6b ^BC^	0.97	186.35 ± 5.6 ^cD^
	NEG-Control	1221.83 ± 36.38 ^A^	84 ± 4.48398 ^cD^	14.55	493.25 ± 51.14 ^aA^

Note: Data are showed as mean ± standard deviation (full range). Identical letters indicate no significant difference (*p* > 0.05); same capital letters and different lowercase letters indicate significant differences (0.01 < *p* < 0.05); different lowercase letters and different capital letters indicate significant differences (*p* < 0.01).

## Supplementary Materials

The following materials are available online at https://www.mdpi.com/2076-2615/10/9/1674/s1, Table S1: The fecal scores of piglets in the first week post challenge; Table S2: PEDV RNA shedding patterns for all challenged piglets over time.
