# Supplementary material for: Evaluation of Cross-Protection between G1a- and G2a-Genotype Porcine Epidemic Diarrhea Viruses in Suckling Piglets
Source: Animals (Basel). 2020 Sep 17;10(9):1674. doi: 10.3390/ani10091674 (PMC7552732; doi:10.3390/ani10091674)
Supplement: Supplementary file 1 [file animals-10-01674-s001.pdf]

Table S1 The fecal scores of piglets in the first week post challenge

	dpc 1	dpc 2	dpc 3	dpc 4	dpc 5	dpc 6	dpc 7	Total
EXP-1a-C-1a								
FS=0	10	10	9	9	9	10	10	67
FS=1	0	0	1	1	1	0	0	3
FS=2	0	0	0	0	0	0	0	0
FS=3	0	0	0	0	0	0	0	0
EXP-1a-C-2a								
FS=0	2	2	1	0	1	1	2	9
FS=1	3	2	0	1	1	1	2	10
FS=2	2	2	3	1	1	2	0	11
FS=3	3	4	5	5	2	0	0	19
EXP-2a-C-2a								
FS=0	10	10	8	8	9	10	10	65
FS=1	0	0	2	2	1	0	0	5
FS=2	0	0	0	0	0	0	0	0
FS=3	0	0	0	0	0	0	0	0
EXP-2a-C-1a								
FS=0	10	9	9	9	10	9	10	66
FS=1	0	1	1	1		1	0	4
FS=2	0	0	0	0	0	0	0	0
FS=3	0	0	0	0	0	0	0	0
POS-1a								
FS=0	2	1	0	0	1	1	1	6
FS=1	1	1	1	1	1	3	2	10
FS=2	1	1	3	2	3	1	1	12
FS=3	6	7	6	6	3	0	0	28
POS-2a								
FS=0	1	1	1	0	0	0	0	3
FS=1	1	1	0	0	0	0	0	2
FS=2	1	2	2	0	0	0	0	5
FS=3	7	5	5	4	0	0	0	21
NEG-Control								
FS=0	10	10	S	9	9	10	10	58
FS=1	0	0	0	1	1	0	0	2
FS=2	0	0	0	0	0	0	0	0
FS=3	0	0	0	0	0	0	0	0

Table S2 PEDV RNA shedding patterns for all challenged piglets over time

	piglets fecal PEDV RNA shedding titer, log_{10} GE/ml						
	dpc 1	dpc 2	dpc 3	dpc 4	dpc 5	dpc 6	dpc 7
EXP-1a-C-1a							
A1	-	-	-	4.22	3.1	-	-
A2	-	-	5.58	5.12	3.15	2.95	-
A3	-	-	4.05	3.15	2.85	-	-
A4	-	-	4.75	4.32	2.88	-	-
A5	-	-	5.01	5.51	4.02	3.75	-
EXP-1a-C-2a							
B1	-	5.01	6.25	6.98	6.55	5.15	4.55
B2	6.53	7.85	D	D	D	D	D
B3	7.51	8.78	7.95	D	D	D	D
B4	-	7.15	6.18	6.99	5.85	5.15	5.1
B5	7.24	7.01	7.18	7.75	8.16	D	D
EXP-2a-C-2a							
C1	-	-	-	-	-	-	-
C2	-	-	-	-	-	-	-
C3	-	-	-	-	-	-	-
C4	-	-	3.5	3.25	-	-	-
C5	3.15	4.01	3.95	3.55	3.05	-	-
EXP-2a-C-1a							
D1	-	-	-	-	-	-	-
D2	-	-	-	-	-	-	-
D3	-	-	-	-	-	-	-
D4	-	4.55	4.2	3.95	-	-	-
D5	-	3.09	-	-	-	-	-
POS-1a							
E1	7.45	8.55	9.01	D	D	D	D
E2	7.01	7.89	7.12	7.01	5.95	5.55	5.15
E3	6.25	6.58	7.12	8.26	D	D	D
E4	6.15	7.01	7.89	8.12	8.1	D	D
E5	6.01	5.12	5.95	5.65	5.01	4.89	5.01
POS-2a							
F1	8.55	9.85	10.55	D	D	D	D
F2	7.58	9.1	10.95	D	D	D	D
F3	8.12	9.96	11.05	D	D	D	D
F4	7.01	8.99	10.51	11.15	D	D	D
F5	9.12	10.89	D	D	D	D	D

NEG-Control							
G1	-	-	-	-	-	-	-
G2	-	-	-	-	-	-	-
G3	-	-	-	-	-	-	-
G4	-	-	-	-	-	-	-
G5	-	-	-	-	-	-	-

Note: D: dead, no sample; -: negative or RNA copies lower than 2.8log10 GE/ml.
